# Supplementary material for: Effect of Catalyst Crystallinity on V-Based Selective Catalytic Reduction with Ammonia
Source: Nanomaterials (Basel). 2021 May 30;11(6):1452. doi: 10.3390/nano11061452 (PMC8230001; doi:10.3390/nano11061452)
Supplement: Supplementary file 1 [file nanomaterials-11-01452-s001.zip › nanomaterials-1228275-supplementary.pdf]

nanomaterials

Supporting information

# Effect of Catalyst Crystallinity on V-Based Selective Catalytic Reduction with Ammonia

**Min Seong Lee [1,2,†], Sun-I Kim [1,†], Myeung-jin Lee [1], Bora Ye [1], Taehyo Kim [1], Hong-Dae Kim [1], Jung Woo Lee [2,*] and Duck Hyun Lee [1,*]**

1 Green Materials and Processes R&D Group, Korea Institute of Industrial Technology, Ulsan 44413, Republic of Korea; qneh5@kitech.re.kr (M.S.L.); sunikim@kitech.re.kr (S.-I.K.); leemj@kitech.re.kr (M.-j.L.); yebora@kitech.re.kr (B.Y.); thkim0215@kitech.re.kr (T.K.); hdkim@kitech.re.kr (H.-D.K.)

2 Department of Materials Science & Engineering, Pusan National University, Busan 46241, Republic of Korea

* Correspondence: jungwoolee@pusan.ac.kr (J.W.L.); Tel.: +82-51-510-2898 (J.W.L.); dulee@kitech.re.kr (D.H.L.); Tel.: +82-52-980-6709 (D.H.L.); Fax: +82-52-980-6669 (D.H.L.)

† These authors contributed equally to this study and should be considered as co-first authors.

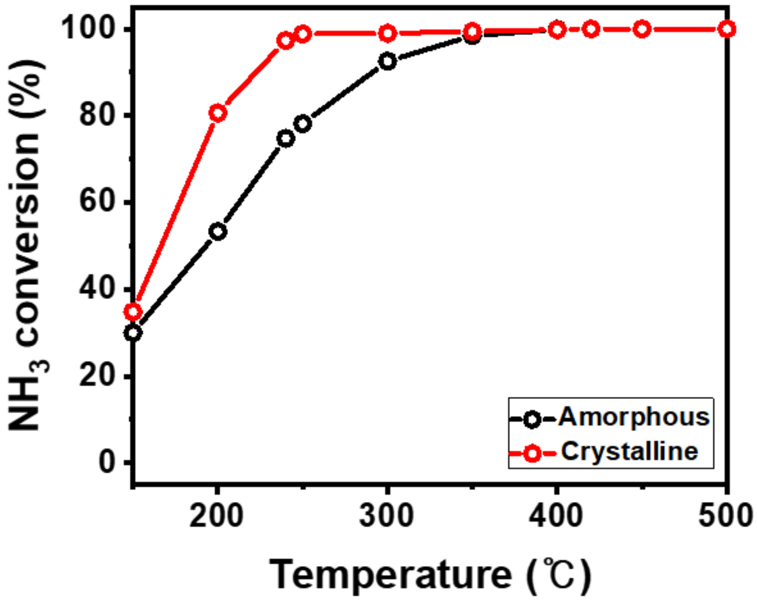


**Figure S1.** NH3 conversion of amorphous and crystalline V2O5-WO3/TiO2 catalyst, which was calculated with equation 3.

NH3 conversion was calculated with equation 3.

$$NH_3 \text{ conversion } (\%) = \frac{NH_{3\ inlet} - NH_{3\ outlet}}{NH_{3\ inlet}} \times 100 \qquad (3)$$

$NH_3$ conversion of crystalline $V_2O_5$-$WO_3/TiO_2$ catalyst is higher than amorphous $V_2O_5$-$WO_3/TiO_2$ catalyst at temperature range 150–350 °C, and both catalysts maintain $NH_3$ conversion rate of 100 above 350 °C. Also, $NH_3$ conversion value is very similar to the $NO_x$ conversion rate.

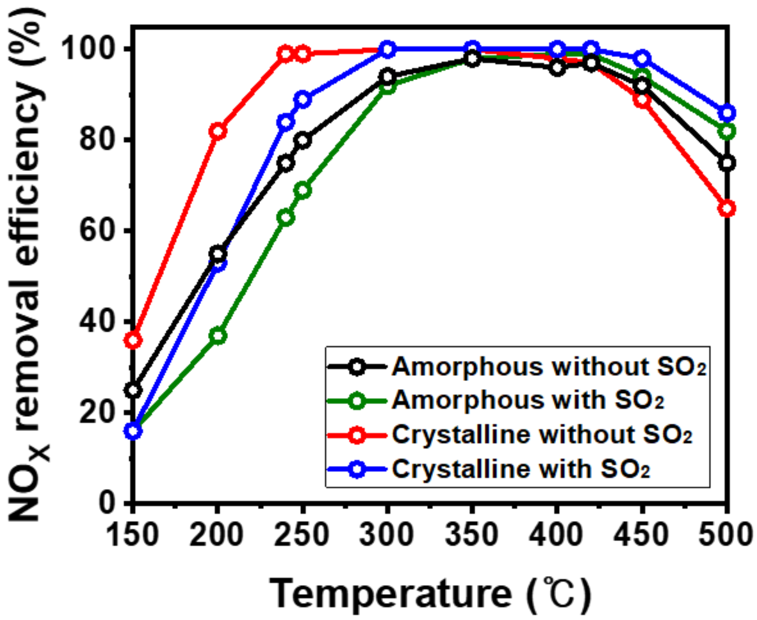


**Figure S2.** $NO_X$ removal efficiency of Amorphous and Crystalline $V_2O_5$-$WO_3$/$TiO_2$ catalyst. Reaction conditions: [NO] & [$NH_3$] = 300 ppm, [$SO_2$] = 0 or 300 ppm, [$O_2$] = 5 vol.%, [GHSV] = 60,000 $h^{-1}$.

We compared the $NO_X$ removal efficiency of amorphous and crystalline $V_2O_5$-$WO_3$/$TiO_2$ catalyst in the gas condition containing $SO_2$. From the result, $NO_X$ removal efficiency of crystalline $V_2O_5$-$WO_3$/$TiO_2$ catalyst is higher than amorphous $V_2O_5$-$WO_3$/$TiO_2$ catalyst, which means that crystalline $V_2O_5$-$WO_3$/$TiO_2$ catalyst is good to SCR reaction regardless of $SO_2$.
